# Editorial: Biomarkers in CKD

**DOI:** 10.3389/fmed.2017.00168

**Published:** 2017-10-10

**Authors:** Magali Araujo, Sonia Q. Doi

**Affiliations:** ^1^Department of Medicine, Georgetown University, Washington, DC, United States; ^2^Department of Medicine, Uniformed Services University, Bethesda, MD, United States

**Keywords:** biomarkers, chronic kidney diseases, diagnosis, progression disease, treatment

**Editorial on the Research Topic**

**Biomarkers in CKD**

The prevalence of chronic kidney disease (CKD) is increasing worldwide and consequently the risks for renal replacement therapies, such as dialysis and transplantation and the incidence of cardiovascular events are also increasing (1, 2). The goal of the present Research Topic, “Biomarkers in CKD” is to highlight the importance of identifying novel and more efficient biomarkers for predicting the decline of glomerular filtration rate (GFR) and the progression of renal disease, as well as to provide tools for more efficient treatments for patients with CKD. We are thankful to all the authors for their invaluable contribution to this topic, and the reviewers for their in-depth service. The collection of papers in this topic demonstrates the complexity of this field and emphasizes new methods and approaches on biomarker discovery with a potential to stratify patients in future clinical studies, help in diagnostics, and reveal new targets for more efficient therapeutic strategies.

Currently, the estimation of GFR by serum creatinine-based equations is the most precise method to estimate the decline of renal function with the progression of kidney disease or the effect of a treatment (3). However, as emphasized by Wilkinson et al., serum creatinine can be affected by a variety of factors that are particularly variable in CKD patients, including muscle mass, dietary protein, and exercise. Therefore, the estimating equations may over- or underestimate GFR in a large population study. The authors highlight the importance of taking in consideration these factors when using the standard formulas for risk assessment of cardiovascular events and mortality in population-based cohorts.

Albuminuria is the classic sensitive marker of early renal dysfunction but excludes many patients that will only have proteinuria when GFR is already significantly decreased. Albuminuria is also utilized as a marker of risk for CKD progression. However, strategies that decrease albuminuria do not always prevent the progression of kidney disease. In this regard, studies have reported various biomarkers that precede changes in albuminuria (4–6). In their comprehensive review of recent clinical studies, Argyropoulos et al. highlight the potential of β2M (beta-2 microglobulin) as a promising marker to assess glomerular and tubular function in a wide spectrum of renal diseases and to predict cardiovascular morbidity and mortality. Prakoura and Chatziantoniou refer to periostin as another candidate. The expression of this protein is low in normal kidneys, increasing with progression of CKD in several animal models or decreasing with regression of the disease upon treatment. These authors also suggest that discoidin domain receptor 1, an integral membrane receptor that is overexpressed in kidney disease, could be a promising therapeutic target for CKD.

The diverse etiological causes and the different rate of progression in patients with CKD have led to the assumption that a multi-biomarker approach has a better potential to provide information with higher accuracy than the analysis of individual biomarkers. The development of new computer-based modeling analyses of transcriptomes and proteomics allow for interpretation of data sets from various sources to identify potential biomarkers in specific population studies. A pilot study conducted by Subasi et al. used mass spectrometry (SELDI-TOF) data to determine patterns of protein expression that predict rapid or slow progression in CKD patients in the African American Study of Kidney Disease and Hypertension (AASK) trial. The authors used a combinatorial Logical Analysis of Data methodology to discriminate the risk groups and to predict the risk for progression of renal disease in that population, demonstrating that this new method outperforms the classic predictor proteinuria, with higher sensitivity, specificity, and accuracy.

In the past two decades, an increasing effort has been applied for the discovery of novel biomarkers, but the potential utility of these markers in the clinical setting is still an area of intense investigation. Therefore, advancing studies on new technology for biomarker discovery is essential to allow the establishment of non-invasive and more accurate markers that can diagnose CKD at its early stages, which can predict or correlate better with the decline of GFR, and which provide more efficient therapeutic targets in the diverse settings of CKD.

## Author Contributions

The authors reviewed manuscripts submitted to the research topic and the final version of this Editorial.

## Conflict of Interest Statement

The authors declare that the research was conducted in the absence of any commercial or financial relationships that could be construed as a potential conflict of interest. The reviewer PS and handling editor declared their shared affiliation.
